# Supplementary material for: Identification of Freezing-Responsive microRNAs and Their Targets in Chinese Jujube by Small RNA and Degradome Sequencing
Source: Int J Mol Sci. 2026 Sep 4;27(17):7912. doi: 10.3390/ijms27177912 (PMC13566890; doi:10.3390/ijms27177912)
Supplement: Supplementary file 1 [file ijms-27-07912-s001.zip › Supplementary Material S2/novel_miR_39.pdf]

Provisional ID	:	novel_miR_39
Score total	:	0.4
Score for star read(s)	:	-1.3
Score for read counts	:	0
Score for mfe	:	0.1
Score for randfold	:	1.6
Score for cons. seed	:	
Total read count	:	112
Mature read count	:	103
Loop read count	:	9
Star read count	:	0

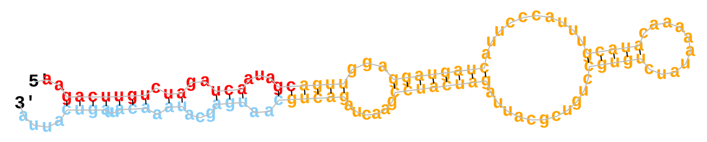

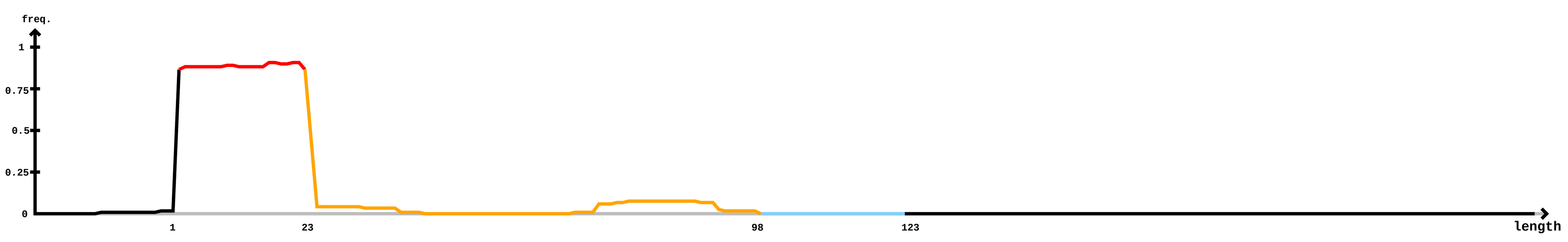

Mature Star

```
5'- uuaaauaacaaaucccucagcaaagacuugucuagaucaauagcaguuggaggaugaucauucccauuugcauacaaaaauaucugugccugucgcauuagaucauccgaacuagacugcaaugagcauaaacauuagucauuaucaacuucacuccauauucuuuuaaguugguucaauaagaagaauccgcuuccuaaguuaguucaaccacgauaggaaugugacacucauguuguuuaugggggauu -3'
```

read	reads	mm	sample
aaaucccucagcaaagacuuguc	1	0	LT-3
gcaaagacuugucuagauca	1	0	LT-3
aagacuugucuagaucaauag	5	0	LT-2
aagacuugucuagaNcaauagc	1	1	LCK-1
aagacuugucuagaucaauagc	41	0	LCK-1
aagacuugucuagaucaauagc	2	0	LT-1
aagacuugucuagaucaauagc	52	0	LCK-2
agacuugucuagaucaauagca	2	0	LT-3
ucuagaucaauagcaguuggagg	1	0	LT-2
caauagcaguuggaggaugauc	2	0	LT-1
caauagcaguuggaggaugauc	1	0	LT-3
agcaguuggaggaugaucauuc	1	0	LT-1
ccugucgcauuagaucauccg	1	0	LT-3
ucgcauuagaucauccgaac	5	0	LCK-3
ucgcauuagaucauccgaacuagacug	1	0	LT-1
cCuuagaucauccgaacu	1	1	LT-2
uuagaucauccgaacuagacug	1	0	JCK-3
